# Recent advances in the immunological understanding of association between tonsil and immunoglobulin A nephropathy as a tonsil‐induced autoimmune/inflammatory syndrome

**DOI:** 10.1002/iid3.248

**Published:** 2019-04-07

**Authors:** Yasuaki Harabuchi, Miki Takahara

**Affiliations:** ^1^ Department of Otolaryngology—Head and Neck Surgery Asahikawa Medical University Asahikawa Japan

**Keywords:** immunoglobulin A, immunoglobulin A nephropathy, tonsil, tonsillar focal disease

## Abstract

**Introduction:**

Immunoglobulin A nephropathy (IgAN) is the most common form of primary glomerulonephritis worldwide. It is well known that upper respiratory tract infections, particularly acute tonsillitis, often worsen IgAN. Recent many clinical studies clearly show that tonsillectomy with steroid pulse therapy is the effective treatments for IgAN patients. Recently, the immunological evidence of association between tonsil and IgAN has been reported.

**Methods:**

In this review, the mechanism underlying the onset of IgAN, as a tonsil‐induced autoimmune/inflammatory syndrome (TIAS), is outlined with the main focus on the authors' research results.

**Results:**

In the tonsils of patients with IgAN, hyperimmune response to the unmethylated deoxycytidyl‐deoxyguanosine oligodeoxynucleotides (CpG‐ODN) take place, resulting in hyperproduction of interferon‐γ. The hyperproduction is followed by both overproduction of mutated IgA via B‐cell activating factor (BAFF)/a proliferation‐inducing ligand (APRIL)‐mediated pathways and overexpression of T‐cell receptor Vβ6, CXCR3, and CX3CR1 on tonsillar T cells. These IgA and T cells home to the kidney via the systemic circulation, resulting in nephritis of IgAN.

**Conclusions:**

Scientific evidence supporting the use of tonsillectomy has gradually accumulated. We hope that many additional researchers will publish new evidence linking the tonsils and kidneys in the future.

## 1 INTRODUCTION

Immunoglobulin A (IgA) nephropathy (IgAN) is the most common form of primary glomerulonephritis worldwide. It is well known that upper respiratory tract infections, particularly acute tonsillitis, often worsen IgAN. Recent many clinical studies clearly show that tonsillectomy with steroid pulse therapy is the effective treatment for IgAN patients,^1^, ^2^, ^3^ and the now the therapy is recommended as a treatment option by the evidence‐based clinical guideline for IgAN.^4^ Therefore, IgAN is now categorized as one of the tonsil‐related diseases, that is defined as “a disease that causes responsive organic or functional injury to other organs away from the tonsils, but with the tonsils as the focus,” and it is a group of diseases in which tonsillectomy is an extremely effective treatment. Although in the past, the terms “focal tonsillitis” or “tonsillar focal infection” were used, today, these are commonly referred to as tonsillar focal diseases, and they encompass an autoimmune and inflammatory disease syndrome that is triggered by the breakdown of immune tolerance to resident bacteria in the tonsils, and is therefore referred to as tonsil‐induced autoimmune/inflammatory syndrome (TIAS).^5^, ^6^ In this short review, the recent advances of the mechanism underlying the onset of IgAN, as a TIAS, are outlined, with the main focus on the authors' research results.

## 2 HISTOLOGICAL BACKGROUNDS OF TIAS

TIAS has a long history. In 650 bc, the relationship between king's disease and dental caries was described in cuneiform documents, and Hippocrates described the relationship between oral disease and rheumatoid arthritis. In the past, the pathogenesis of TIAS was thought to be a type of sepsis that spread from a bacterial infection at the focus itself, or from toxins from the bacteria. Because of these characteristics, rheumatic diseases that occur after β‐streptococcal infection, such as glomerular nephritis, rheumatic fever, acute rheumatoid arthritis, endocarditis, and myocarditis, were considered tonsillar focal diseases until the beginning of the 20th century. However, as the number of β‐streptococcal infections decreased with the widespread use of antibiotics, aspects of these secondary diseases also began to change.

Recently, tonsillectomy has been reported to be extremely effective for palmoplantar pustulosis,^7^ pustulotic arthro‐osteitis, 7 and IgAN,^1^, ^2^, ^3^ and the tonsil has been established as the focus of these diseases. In addition, there are a large number of reports that tonsillectomies are also effective^8^ for psoriatic diseases,^9^, ^10^, ^11^ and IgA vasculitis,^12^ reactive arthritis,^13^ periodic fever, aphthous stomatitis, and pharyngitis and cervical adenitis syndrome.^14^

## 3 THE PALATINE TONSILS AS PART OF THE MUCOSAL IMMUNE SYSTEM OF THE UPPER RESPIRATORY TRACT

**Figure 1:**
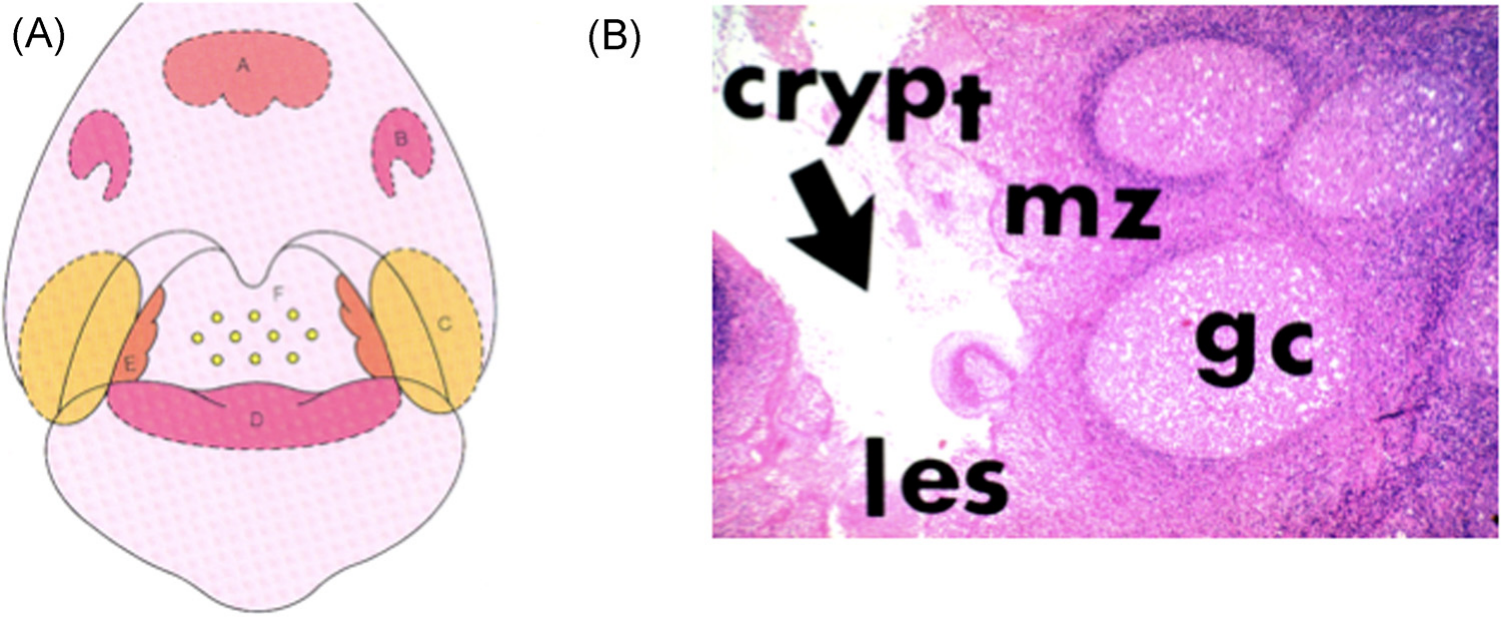
Location and histological picture of tonsils composing Waldeyer's ring. A, Schematic figure of Waldeyer's ring (A, pharyngeal tonsil; B, tubal tonsil; C, palatine tonsil; D, lingual tonsil; E, lateral lymphatic band; F, lymphoid follicle on posterior wall of oropharynx). B, Structure of palatine tonsil (les, lymphoepitherial symbiosis; mz, mantle zone; gc, germinal center)

Generally, “tonsils” refer to the palatine tonsils, a pair of left and right lymphatic tissues located in the oropharynx. The tonsils (palatine tonsils) together with the pharyngeal tonsils, tubal tonsils, lingual tonsils, and lymphoid follicles of the lateral pharyngeal wall form an annular group of lymphoid tissues that are present in the pharynx and are collectively called Waldeyer's tonsillar ring (Figure 1A). The nasal cavity, due to its anatomical position in the first part of the upper respiratory tract, defends against the invasion of bacteria and viruses into the oral cavity, and functions as an immune organ as part of the mucosa‐associated lymphoid tissue, much like Peyer's patches in the small intestine.

**Figure 2:**
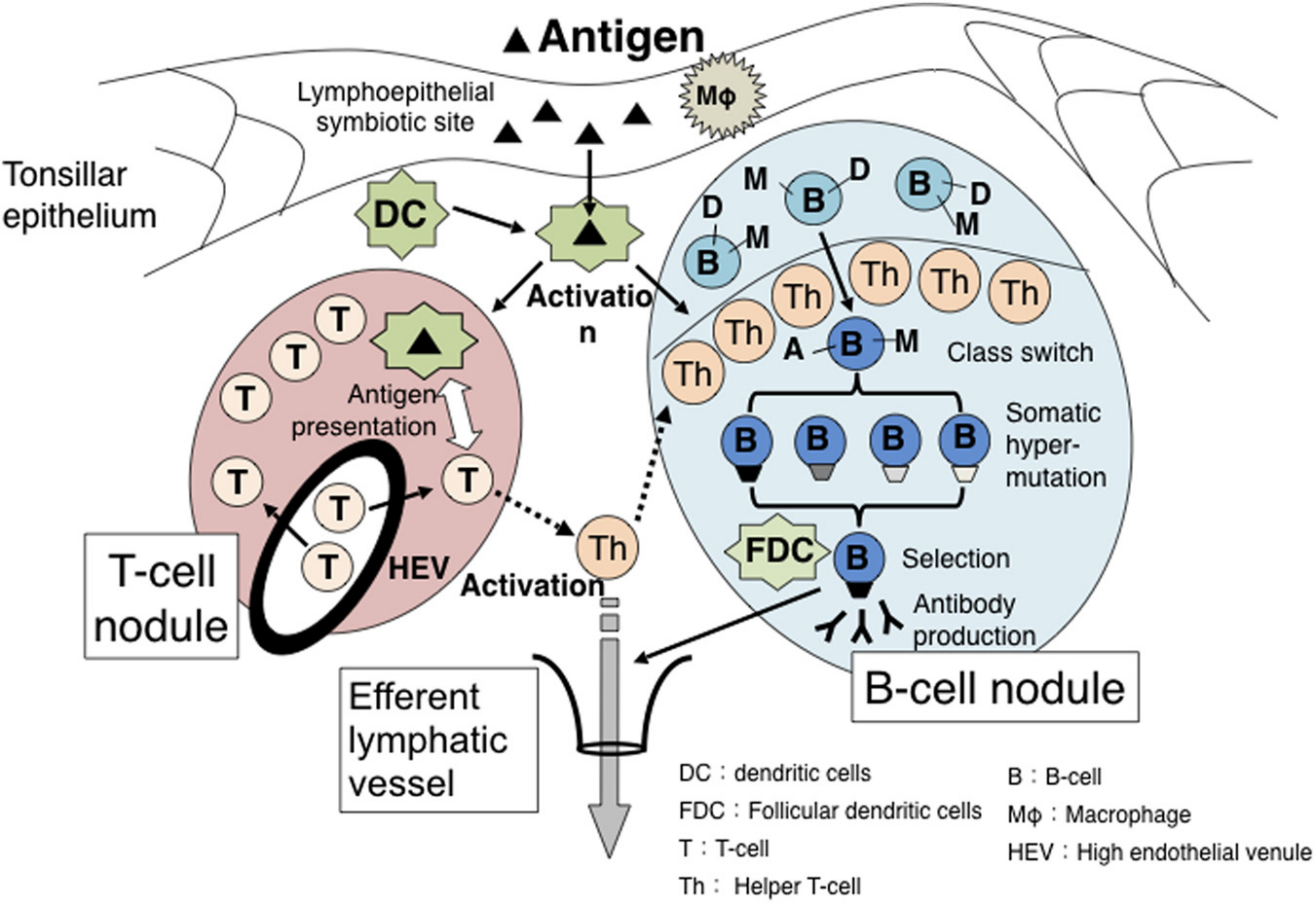
Immunological reaction against antigens in the tonsil

The palatine tonsils are composed of B‐cell‐dominant lymphocytes and a few myeloid cells; however, unlike normal peripheral lymph nodes, there are no afferent lymphatic vessels. The surface of the palatine tonsils is covered with nonkeratinizing squamous epithelium, which branches deeply into the tonsils to form crypts. Because of this crypt structure, the tonsil has six times the surface area of the entire pharyngeal mucosa. The blind end of the crypt, where the crypt epithelium and tonsil parenchyma are mixed, is called the lymphoepithelial symbiosis, and its structure is characteristic of the tonsils (Figure 1B). Antigen‐presenting cells, such as M cells (membranous epithelial cells) and dendritic cells, as well as memory B cells are distributed in the lymphoepithelial symbiosis site and are thought to be the starting point for antigen recognition in the tonsils. The deep part of the lymphoepithelial symbiosis is the tonsil parenchyma, which, like peripheral lymph nodes, is composed of lymphoid follicles and interfollicular areas. The interfollicular area is a T‐cell‐dependent region, where mainly T cells are distributed. Dendritic cells complement their antigens at lymphoepithelial symbiosis sites, and antigen presentation occurs on naive T cells flowing from the high endothelial venules. Lymphoid follicles consist of a mantle zone surrounding a germinal center. A characteristic of the mantle zone is that it develops towards the crypt side and, due to its shape, is called the “cap zone,” and the germinal center is located in the deep regions of the mantle zone. Small mature B cells are present in the mantle zone, and follicular helper T cells and macrophages that are distributed like bands on the mantle zone side are present in the germinal center. Through the activities of these cells, B cells are activated, and they undergo class switching to differentiate into immunoblasts. Then, diverse antibodies are generated by somatic hypermutation, and the cells differentiate into memory B cells through modification of follicular dendritic cells (Figure 2).^15^, ^16^

Tonsillar lymphocytes show proliferative responses and active DNA synthesis even when cultured in the absence of mitogens^17^ and have the ability to produce immunoglobulins such as IgG and IgA^18^ as well as a wide variety of cytokines.^19^, ^20^, ^21^ Therefore, activated lymphocytes are abundant in the tonsils, which is unlike in the peripheral blood and peripheral lymph nodes. In addition, tonsillar lymphocytes exhibit an activated response when stimulated with pathogenic bacteria, such as pneumococci and *Haemophilus influenza*, or with antigens produced by other pathogens, such as mites and viruses, invading from the upper respiratory tract,^22^ but they do not show an activated response to bacteria indigenous to the tonsils, such as α‐streptococci.^23^ When the tonsils are locally sensitized to tetanus vaccine, many cells that produce specific antibodies appear in the tonsils, and these antibodies can then be detected in serum or pharyngeal secretions.^24^ These findings suggest that the tonsils function by sending memory B cells and immunoglobulin precursor cells to the pharynx and throughout the body via antigen activation. Thus, it is thought that the tonsils function as the induction site in the mucosal immune system of the upper respiratory tract.

## 4 IGA OVERPRODUCTION IN TONSILS OF PATIENTS WITH IGAN

It is commonly accepted that serum IgA is strongly involved in the pathogenesis of IgAN, but a source of serum IgA has not been defined in the past. However, on the basis of the recent works, the tonsils are thought to be a source organ of serum IgA in patients with IgAN. In patients with IgAN, serum IgA levels decrease after tonsillectomy.^25^The number of multimeric IgA‐producing cells with a J chain is increased in the tonsils with IgAN,^26^, ^27^ and when the tonsillar lymphocytes are cultured under mitogenic stimulation, the production of polymeric IgA is enhanced.^28^ Furthermore, in the germinal centers of the tonsil tissues in patients with IgAN, the number of CD5‐positive B cells involved in IgA1 production increase.^29^ These findings suggest that the tonsils are a source of serum IgA in patients with IgAN.^30^

Normally, the tonsillar immune reaction acts against pathogenic bacteria, whereas the immune tolerance network reacts against indigenous bacteria that compose of normal bacterial flora in the tonsil, such as α‐streptococci, so that no activation response occurs.^23^ However, in the tonsils of patients with IgAN, immune tolerance fails, and an excessive immune response to indigenous bacterial and bacterial DNA occurs.

A DNA sequence commonly present in all bacteria (unmethylated deoxycytidyl‐deoxyguanosine oligodeoxynucleotides (CpG‐ODN) is a ligand for toll‐like receptor 9 (TLR9) and induces an innate immune response. Goto et al^31^ investigated the possibility that this unmethylated CpG‐ODN is an antigen that triggers an immune response. In vitro study showed that tonsillar mononuclear cells of IgAN patients showed significantly higher productions of IgA and interferon‐γ (IFN‐γ) in response to CpG‐ODN. This suggests that hyperimmune response to CpG‐ODN, that is, microbial DNA, may be involved in tonsils of IgAN patients. Goto et al^31^ further found the increase in spontaneous IFN‐γ and IgA productions of tonsillar mononuclear cells as found elsewhere.^32^ Furthermore, tonsillar mononuclear cells of IgAN patients showed significantly higher production of B‐cell activating factor (BAFF), which belongs to the tumor necrosis factor family and promotes immunoglobulin production independent of T cells. Subsequently, Takahara et al^33^ focused on aberrant expression of a proliferation‐inducing ligand (APRIL), which functions like BAFF to activate B cells independent of T cells. In the tonsillar mononuclear cells of patients with IgAN, CpG‐ODN stimulation resulted in increased production of APRIL and an increase in transmembrane activator and transmembrane activator and calcium‐modulating cyclophilin ligand interactor (TACI) expression, which is an APRIL receptor. Further, culture of tonsillar B cells from patients with IgAN in the presence of CpG‐ODN and APRIL showed that IgA production was significantly enhanced. In addition, serum APRIL levels were significantly higher in the IgAN group than in the habitual tonsillitis group and was significantly decreased after tonsillectomy. Muto et al^34^ also reported that the enhanced expression of APRIL in tonsil tissue (germinal center) and the increase in TLR9 expression are related and that it is clinically associated with worsening urinalysis parameters.

These findings suggest that the tonsils of patients with IgAN are excessively reactive to bacterial DNA (CpG‐ODN) and IFN‐γ, resulting in overproduction of BAFF and APRIL, and subsequent overproduction of IgA via overexpression of its receptors TACI. Goto et al 31 and Takahara et al 33 detected an increase in the expression of TLR9, which is a ligand for CpG‐ODN, in the tonsillar B cells of patients with IgAN, and Sato et al^35^ reported the high efficacy of tonsillectomy plus steroid pulse therapy in groups of patients with high tonsillar TLR9 expression. In experiments using animal models, Suzuki et al^36^ reported that elevated serum IgA levels and increased IgA deposition on renal glomeruli resulted from intranasal administration of CpG‐ODN to IgAN model mice (ddy mice). It was reported that polymorphism of the TLR9 gene is involved in the pathology of IgAN,^36^ which suggests that the CpG‐ODN‐TLR9 pathway is deeply involved in its pathology.

## 5 PRODUCTION OF ABERRANTLY GLYCOSYLATED IGA IN THE TONSILS

The IgA produced by patients with IgAN is aberrantly glycosylated, and there is an increase in aberrantly glycosylated IgA1 in the blood and glomeruli of IgAN patients.^37^, ^38^, ^39^ In the tonsillar lymphocytes of IgAN patients, increased production of aberrantly glycosylated IgA1 in vitro^40^, ^41^, ^42^ and the decreased expression of glycosyltransferases for IgA1,^43^ which likely contributes to the glycosylation deficiency, were observed, suggesting that the site where the aberrantly glycosylated IgA1 is produced may be located in the tonsil. It is known that B‐cell lymphoma 2 (Bcl‐2), a molecule that suppresses apoptosis in B cells, enhances the production of aberrantly glycosylated IgA and promotes IgA deposition in renal glomeruli.^44^ As BAFF promotes the expression of Bcl‐2 in B cells,^45^ this suggests that BAFF overproduction induced by CpG‐ODN is involved in both the quantitative and qualitative abnormalities of IgA observed in IgAN patients.

## 6 HOMING OF TONSILLAR T CELLS TO THE KIDNEY

It has been reported that, in autoimmune disease target organs, among the 20 types of T‐cell receptor (TCR) Vβ family members, there is an increase in T cells with a specific TCR Vβ. Nozawa et al^46^ analyzed the repertoire of tonsillar T cells in patients with IgAN, and found that the expression of TCR Vβ6 is increased in the tonsillar T cells of patients with IgAN. In addition, when tonsillar lymphocytes were stimulated with the *Haemophilus parainfluenza* antigen, we observed an increase in TCR V6‐positive T cells. When the expression of TCR Vβ6 and Vβ8 in peripheral blood T cells was investigated, Nozawa et al^46^ found that its expression in the IgAN group was increased and was decreased after tonsillectomy. Kidney‐infiltrating T cells in patients with IgAN are known to express high levels of TCR Vβ6 and Vβ8,^47^ which suggests that the TCR Vβ6‐positive tonsillar T cells selectively induced by *H. parainfluenza* may be involved in the development of nephritis in the kidney through the systemic circulation.

Reports on the production of chemokines in glomeruli or the interstitium and the homing of inflammatory cells to kidney tissue are limited. Segerer et al^48^ reported that CXCR3‐positive cells predominantly infiltrated the renal tubule interstitium in patients with IgAN and showed a correlation between the degree of invasion and the decrease in renal function. Takahara et al^49^ analyzed the expression of chemokine receptors in the tonsils and found that CXCR3 expression was enhanced in the tonsillar T cells of patients with IgAN.

CX3CR1 is a chemokine receptor expressed in CD8^+^ T cells and NK cells, and its ligand is fractalkine (CX3CL1). Fractalkine is expressed in vascular endothelial cells, and its binding induces vasculitis.^50^ Otaka et al^51^ showed that the proportion of CD8^+^ CX3CR1^+^ cells in the tonsillar mononuclear cells of patients with IgAN was increased and was significantly increased by stimulation with CpG‐ODN. Similarly, the proportion of CD8^+^ CX3CR1^+^ cells in the population of peripheral blood mononuclear cells in patients with IgAN was also increased, and after tonsillectomy, this proportion was significantly decreased, together with the disappearance of hematuria. Furthermore, hematuria in patients with IgAN was correlated with CX3CR1 expression in the peripheral blood mononuclear cells and fractalkine expression in the renal glomeruli. Therefore, there is a possibility that the tonsillar CD8+ CX3CR1+ cells which invade the glomeruli express its ligand fractalkine, causing vasculitis.

## 7 ONSET OF THE MECHANISM OF IGAN, WITH THE TONSILS AS THE FOCUS

**Figure 3:**
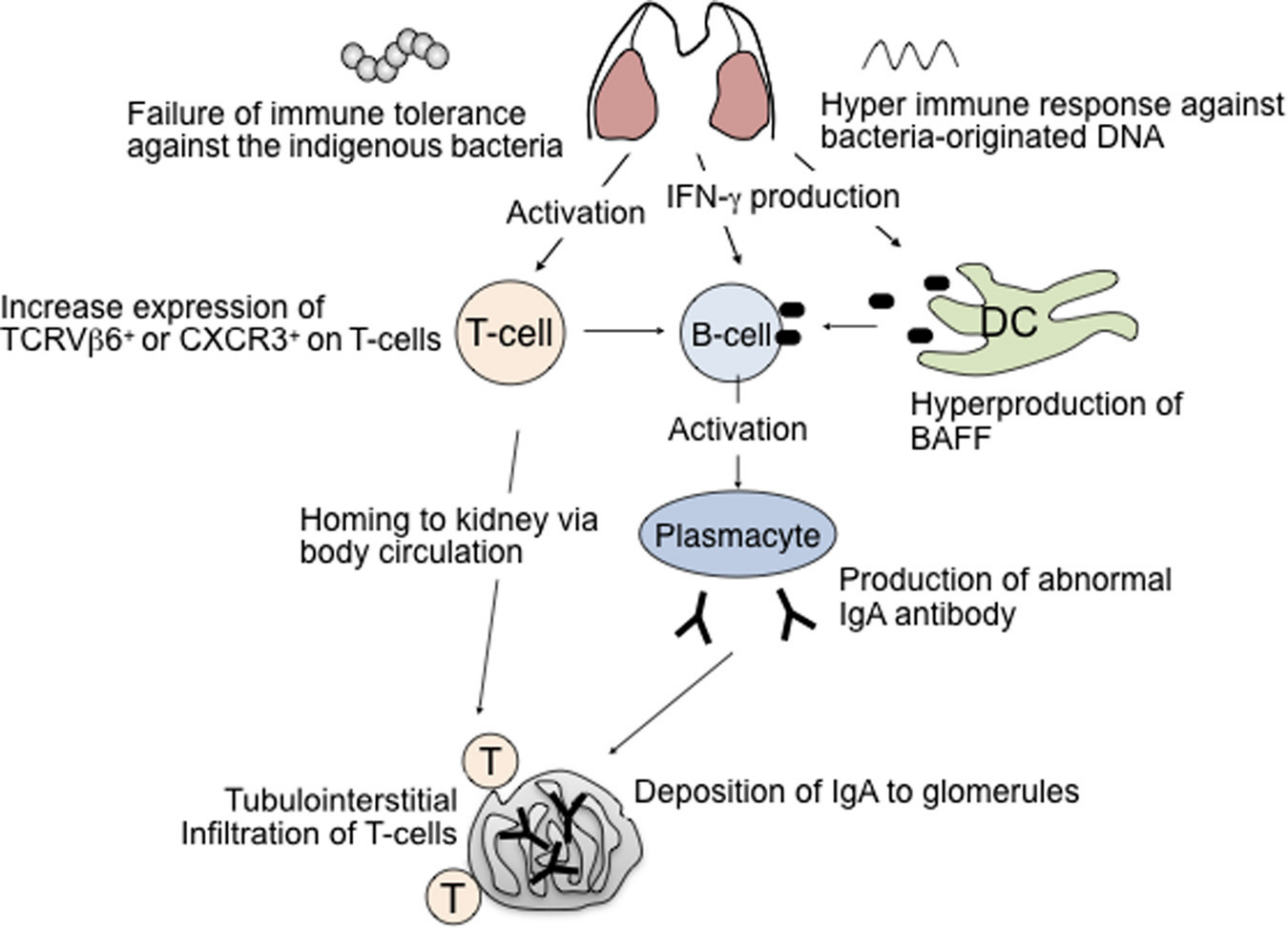
Mechanism of the onset of IgA nephropathy, with the tonsil as the focus. BAFF, B‐cell activating factor; APRIL, A proliferation‐inducing ligand; DC, dendritic cell; IFN‐γ, interferon‐γ; IgA, immunoglobulin A

The pathogenesis of IgAN with the tonsils as the focus was examined based on the results of previous studies (Figure 3). In the tonsils of patients with IgAN, mutated IgA is overproduced via BAFF‐ and APRIL‐mediated T‐cell‐independent pathways triggered by a hyperimmune response (due to disruption of immune tolerance) to the unmethylated CpG‐ODN frequently present in microbial DNA. Meanwhile, in response to this excessive response to unmethylated CpG‐ODN, TCR Vβ 6 on T cells and chemokine receptors with renal tissue affinity (CXCR3 and CX3CR1) are also overexpressed via IFN‐γ. In addition, homing to the kidney via the systemic circulation is thought to be involved in tissue injury.

## 8 CONCLUSION

The efficacy of tonsillectomy for IgAN has been clinically shown by a recent randomized controlled trials,^52^ and it has also been published as a recommended treatment in IgAN clinical practice guidelines.^4^ Recently, scientific evidence supporting the use of tonsillectomy has gradually accumulated, and this treatment is being developed not only in Asia^53^, ^54^ but also in Europe and the United States.^30^, ^55^ We hope that many additional researchers will publish new evidence linking the tonsils and kidneys in the future.
